# A Site-Specific Integrative Plasmid Found in *Pseudomonas aeruginosa* Clinical Isolate HS87 along with A Plasmid Carrying an Aminoglycoside-Resistant Gene

**DOI:** 10.1371/journal.pone.0148367

**Published:** 2016-02-03

**Authors:** Dexi Bi, Yingzhou Xie, Cui Tai, Xiaofei Jiang, Jie Zhang, Ewan M. Harrison, Shiru Jia, Zixin Deng, Kumar Rajakumar, Hong-Yu Ou

**Affiliations:** 1 State Key Laboratory for Microbial Metabolism and School of Life Sciences & Biotechnology, Shanghai Jiaotong University, Shanghai, China; 2 Department of Laboratory Medicine, Huashan Hospital, Shanghai Medical College, Fudan University, Shanghai, China; 3 Key Laboratory of Industrial Microbiology, Ministry of Education, Tianjin University of Science and Technology, Tianjin, China; 4 Department of Infection, Immunity and Inflammation, University of Leicester, Leicester, United Kingdom; University of Manchester, UNITED KINGDOM

## Abstract

Plasmids play critical roles in bacterial fitness and evolution of *Pseudomonas aeruginosa*. Here two plasmids found in a drug-resistant *P*. *aeruginosa* clinical isolate HS87 were completely sequenced. The pHS87b plasmid (11.2 kb) carries phage-related genes and function-unknown genes. Notably, pHS87b encodes an integrase and has an adjacent *tRNA*^*Thr*^-associated attachment site. A corresponding integrated form of pHS87b at the *tRNA*^*Thr*^ locus was identified on the chromosome of *P*. *aeruginosa*, showing that pHS87b is able to site-specifically integrate into the 3’-end of the *tRNA*^*Thr*^ gene. The pHS87a plasmid (26.8 kb) displays a plastic structure containing a putative replication module, stability factors and a variable region. The RepA of pHS87a shows significant similarity to the replication proteins of pPT23A-family plasmids. pHS87a carries a transposon Tn*6049*, a truncated insertion sequence ΔIS*1071* and a Tn*402*-like class 1 integron which contains an *aacA4* cassette that may confer aminoglycoside resistance. Thus, pHS87b is a site-specific integrative plasmid whereas pHS87a is a plastic antibiotic resistance plasmid. The two native plasmids may promote the fitness and evolution of *P*. *aeruginosa*.

## Introduction

Plasmids are an essential factor driving bacterial evolution. Plasmid-encoded functions such as virulence, resistance, metabolism and/or other advantageous functions can promote bacterial fitness [1]. *Pseudomonas aeruginosa* is an important opportunistic pathogen responsible primarily for hospital- and cystic fibrosis-associated infections. Plasmids have been shown to confer advantageous traits upon *P*. *aeruginosa* clinical isolates [2].

Certain plasmids can insert into chromosomes site-specifically [3], contributing to chromosomal mosaicism together with other integrative mobile elements such as pathogenicity islands, integrative and conjugative elements, and prophages in bacteria [1]. Many of the integrative elements have been found to be incorporated into tRNA gene sites of the host chromosome. Plasmid pKLK106 and pKLC102 that coexist as free plasmids and genomic islands are among the very few cases reported of integrative plasmids in *P*. *aeruginosa* [4]. Plasmid pKLC102 that can recombine within the 3′ end of the *tRNA*^*Lys*^ gene has been shown to be phage-related and carry determinants for host tropism and virulence which may allow evolution of the chromosome and genomic island to be traced [4].

Antibiotic resistance is one of the critical issues threatening public health. Plasmids are the most common carriers of antibiotic resistance genes. It has been commonly seen that antibiotic resistance in *P*. *aeruginosa* was correlated with the presence of IncP group conjugative plasmids [5, 6]. Moreover, many resistance plasmids are being detected in *P*. *aeruginosa* [2]. Many of the resistance genes are found embedded in or associated with mobile elements such as transposons, integrons, and IS elements [7–15]. In addition, apart from the genomic island-encoded virulence [16], *P*. *aeruginosa* plasmid-mediated pathogenicity was revealed [17]. Plasmids in *P*. *aeruginosa* also are involved in other fitness traits [18]. However, to our knowledge, no plasmid has been seen in completely sequenced *P*. *aeruginosa* genomes to date (http://www.ncbi.nlm.nih.gov/genome), and only a limited number of *P*. *aeruginosa* plasmids have been fully sequenced and analysed.

Here we sequenced two novel, native plasmids found in a *P*. *aeruginosa* clinical isolate. One is a site-specific integrative plasmid with the size of 11 kb that may drive the plasticity of the genome and has potential to serve as a genetic tool, while another with the size of 26 kb carries mobile genetic elements and an aminoglycoside-resistant gene that may enhance the fitness of the strain. The two plasmids may confer advantageous traits upon and promote evolution of this clinical isolate.

## Materials and Methods

### Ethics and consent

Because this study was an observational study by using human sputum samples for the *ex vivo* experiments and the sampling of patient’s sputum was a routine work in hospital treatment, verbal informed consent was obtained from all volunteers. The bacteria samples and data sheets were anonymized. This study protocol including verbal informed consent procedure was approved by the ethics committee of the School of Life Sciences & Biotechnology, Shanghai Jiaotong University, China.

### Strain

*P*. *aeruginosa* HS87 was isolated from the sputum of an inpatient in Shanghai, China. *P*. *aeruginosa* HS87 was tested resistant to gentamicin, tobramycin, netilmicin and ciprofloxacin, but sensitive to amikacin, tazocin, meropenem, aztreonam and ceftazidime. The HS87 strain was the only strain found to harbour plasmids among twenty-five *P*. *aeruginosa* clinical isolates collected in a hospital in Shanghai.

### Plasmid preparation, sequencing and analyses

Plasmids were prepared using a Plasmid Midi Kit (Qiagen). Plasmids were sequenced and assembled by the Sanger method. Gaps were closed by standard Sanger sequencing of PCR products. Open reading frames (ORFs) were detected based on the prediction results of Glimmer 3 [19], GeneMark [20], and the translation initiation sites of certain ORFs were modified with Prodigal [21]. ORFs were annotated by BLAST against NCBI protein database (see S1 Table and S2 Table). Sequences for comparison were retrieved from NCBI. Phylogenetic tree was constructed by MEGA 5.0 [22] with neighbour-joining method and 1,000 bootstrap replicates, based on MUSCLE [23] alignment.

### Integration of pHS87b

Homologous attachment sites were detected by Blastn. Presence of genomic island (GI) at the *tRNA*^*Thr*^ site was examined by tRIP-PCR (tRNA site interrogation for pathogenicity islands, prophages and other GIs) [24] using primers thr_U and thr_D (S3 Table). Amplification of 16S rRNA gene was performed as a control using universal primers. Attachment site on pHS87b (*attP*) was amplified using primers attP_U and attP_D (S3 Table). Junctions and orientation of the GI form of pHS87b were examined using the combinations of one of the chromosomal primers (thr_U or thr_D) and one of the plasmid primers (attP_U or attp_D). The GI occupying the *tRNA*^*Thr*^ site was amplified via long PCR using thr_U and thr_D. All amplicons were sequenced using Sanger method except the long PCR product which was digested by *Bam*HI, *Bgl*II, *Cla*I or *Sma*I.

In addition, the integration of pHS87b was further confirmed by Southern blotting assays. Probes specific to the chromosome and the pHS87b plasmid were amplified by PCR, with primers pchG-SBF/R targeting the *pchG* gene on the chromosome and int-SBF/R targeting the pHS87b integrase gene *orf2* (S3 Table), respectively. Southern blotting was performed by using DIG Hybridization Detection Kit I (Mylab Co., Beijing, China), according to its user manual.

### Quantitative PCR for examination of *pHS87b* integration frequency

Quantitative real-time PCRs were performed to quantify the abundance of the empty *tRNA*^*Thr*^ site and the circular as well as integrated forms of pHS87b with ABI 7500 Fast Real-Time PCR System (Thermo Fisher Scientific) and HieffTM qPCR SYBR® Green Master Mix (Yeasen). 2 ng of the total DNA of *P*. *aeruginosa* HS87 was used as template in 20 μl reaction volume. The empty site was detected by primers attB-qF and attB-qR targeting the unoccupied chromosomal attachment site (*attB*) (S3 Table). The circular and integrated forms of pHS87b were detected by primers attP-qF and attP-qR targeting the plasmid attachment site (*attP*) (S3 Table), and primers attB-qR and attP-qR targeting the right direct repeat of the integrated pHS87b (*attR*) (S3 Table), respectively. The *gyrB* gene, a single copy chromosomal gene encoding ATPase domain of DNA gyrase, was amplified with primers gyrB-qF and gyrB-qR as the endogenous reference (S3 Table).

### Accession numbers

Sequences were deposited in GenBank under the accession numbers KR106190 for pHS87a and KR106191 for pHS87b.

## Results and Discussion

### Plasmid sequencing

Two circular plasmids were assembled. Plasmid pHS87a is 26,825 bp in length with a G+C content of 62.92%, while pHS87b is 11,242 bp with a G+C content of 60.71%. Annotation revealed 31 and 16 ORFs in pHS87a (S1 Table) and pHS87b (S2 Table), respectively.

### General features of pHS87b

**Fig 1:**
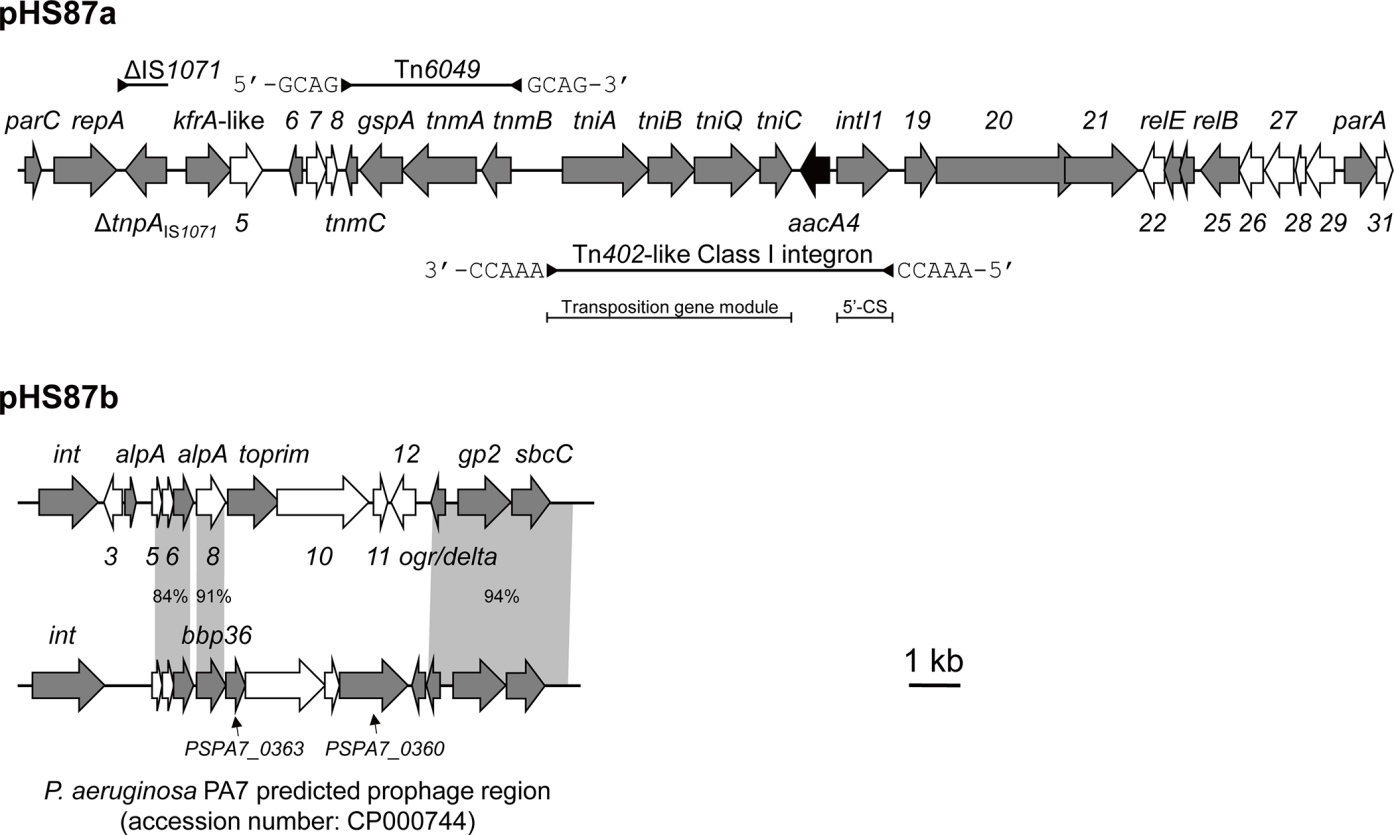
Schematic map of the native plasmid pHS87a and pHS87b found in the *P*. *aeruginosa* clinical isolate HS87. Mobile elements are indicated by the lines above or below the sequence. Antibiotic resistance gene is black; genes with predicted functions are grey; function unknown genes are white. The numbers are *orf* numbers of the function unknown genes. The black triangles facing each other at the termini of a line indicate inverted repeats (IRs) of the element. The single triangle appearing on the line of ΔIS*1071* means it is a truncated element. Direct repeats flanking the elements are shown by corresponding letters. Syntenic regions are linked by grey parallelograms or squares with identities shown. The schematic is drawn to scale.

The native plasmid pHS87b has two regions (*orf5 –orf8* and *orf13 –orf16*) that are nearly identical to the pieces located within a prophage (coordinate: 366452 to 386384) of *P*. *aeruginosa* PA7 [25] (GenBank accession no. CP000744) (Fig 1), which was predicted by *Phage_finder* [26]. The counterparts encode phage-related transcriptional factors, a capsid protein and an exonuclease. The rest of the regions show no synteny to any of the characterised sequences to date. Those regions encode a putative integrase, a TOPRIM domain-containing protein and many other function unknown proteins. No known replication genes were found. The TOPRIM domain-containing protein and some function unknown proteins might be involved in replication. The plasmid was mapped with the data from the NCBI and Human Microbiome Project (HMP) (S1 Fig). The plasmid generally possesses no coverage in the HMP resource and limited coverage in the NCBI database. However, a 45-bp region was highly covered in both resources and the whole plasmid was found syntenic to some *P*. *aeruginosa* chromosomal regions, which will be discussed in the next section.

### Integration of pHS87b

**Fig 2:**
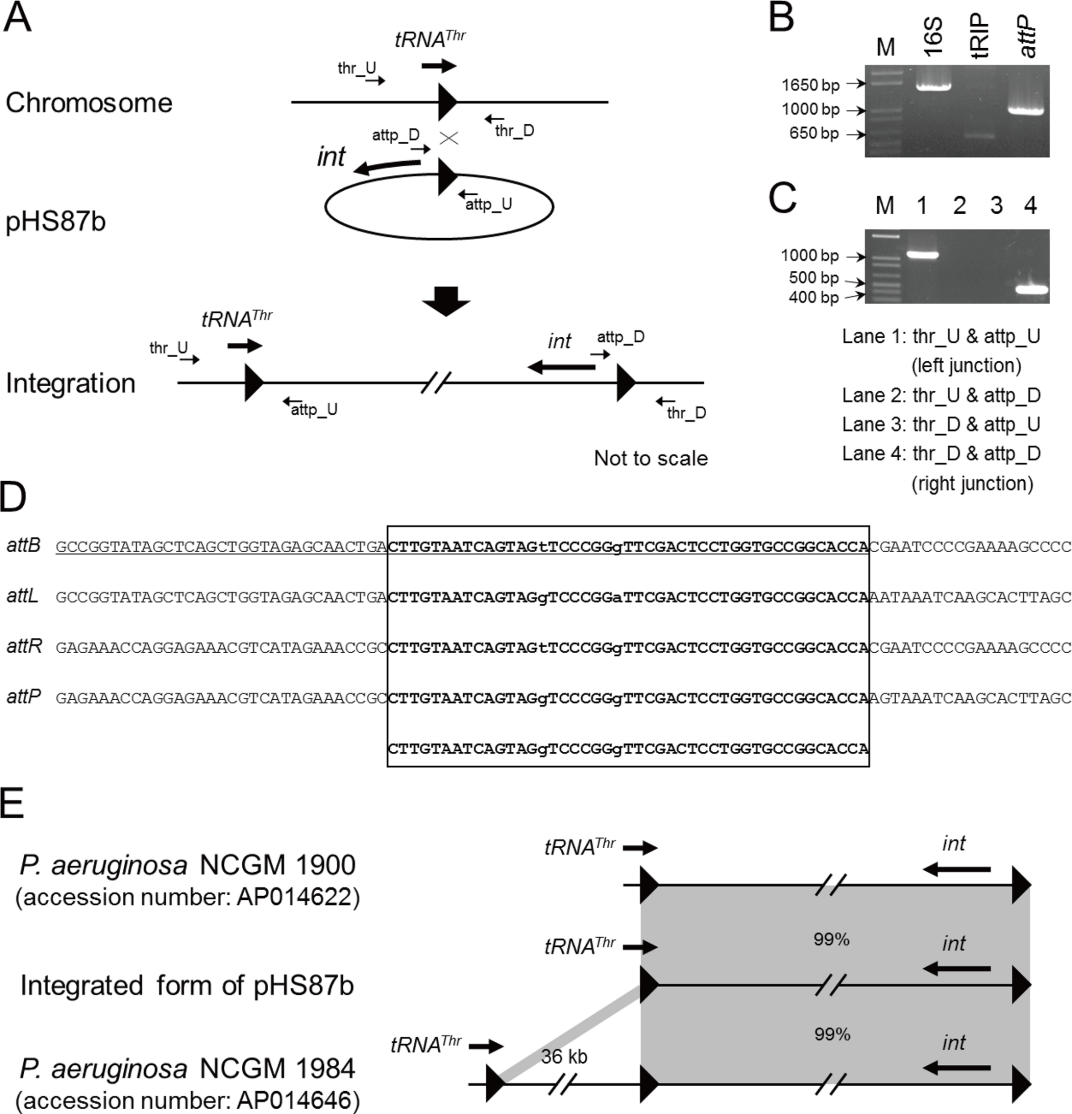
Site-specific integration of pHS87b in to the *tRNA*^*Thr*^ gene site. (A) Integration schematic. Black triangles indicate homologous attachment regions and direct repeats. Genes are indicated by thick arrows. Primers are shown as thin arrows. (B) tRIP-PCR at the assay *tRNA*^*Thr*^ gene site showed a faint band. Amplification of 16S rRNA gene and attachment site of pHS87b was also performed. (C) Amplification of junctions of the integrated pHS87b. (D) Sequencing confirmed the integration of pHS87b into the *tRNA*^*Thr*^ gene site. The *tRNA*^*Thr*^ gene sequence is underlined. The block highlights the homologous region. (E) Alignment of the integrated form of pHS87b with the syntenic chromosomal regions of *P*. *aeruginosa* NCGM 1984 and NCGM 1900. The schematic is not drawn to scale.

Plasmid pHS87b encodes a putative integrase, significantly related to the bacteriophage P4 integrase family, suggesting pHS87b could be integrative (Fig 2A). Most of the integrase-mediated recombination has been shown to be site-specific, which requires an attachment site adjacent to the integrase gene on the circular extra-chromosomal element and a homologous attachment site at the insertion locus, usually within the chromosome [27]. The site-specific recombination mediated by P4 integrase could be both integrative and excised. Blast of the nucleotide sequence of pHS87b against the chromosome sequence of the reference strain *P*. *aeruginosa* PAO1 [28] (GenBank: AE004091) revealed that a 45-bp region upstream of the putative integrase gene (*int*) was identical to the 3’-end of a *tRNA*^*Thr*^ gene (*PA5160*.*1*), which explains the high coverage of this region in the NCBI and HMP resources (S1 Fig). The *tRNA*^*Thr*^ gene has only one copy in *P*. *aeruginosa*. Moreover, tRIP assay at the *tRNA*^*Thr*^ locus resulted in a faint band matching the empty site (Fig 2B), indicating the site was partly occupied by a genomic island (GI) in the population. The tRIP assay is a method to detect tRNA-associated GIs using a pair of primers (e.g. thr_U and thr_D) targeting the upstream and downstream of a tRNA gene [24]. If the tRNA site is empty, there will be only one band with a size corresponding to the empty site. Otherwise, negative or weak amplification of the empty site will be seen depending on the GI integration frequency; meanwhile, a larger band may also appear if the GI size is within the amplification range. To check whether the GI is the integrated form of pHS87b, we managed to amplify the junctions using the primer pairs with one on the chromosome and another on the plasmid (Fig 2C). The orientation of the integration was consistent with the proposed scenario. Meanwhile, the GI was amplified by long PCR with a band having similar size to pHS87b. The restriction profile of the amplicon generated by *Bam*HI, *Bgl*II, *Cla*I, and *Sma*I was in accord with the predicted restriction patterns of the integrated form of pHS87b (S2 Fig).

Finally, sequencing of the junctions and the unoccupied site confirmed that site-specific integration of pHS87b occurred at the *tRNA*^*Thr*^ locus, splitting the attachment site into two direct repeats (Fig 2D). Here, attachment sites (*att*) simply refer to the 45-bp homologous regions. Sequencing of the faint band mentioned above confirmed the presence of attachment site (*attB*) at the empty *tRNA*^*Thr*^ locus in this strain and that the *attB* is near-identical to the attachment site (*attP*) on pHS87b. In addition, direct repeats (*attL* and *attR*) flanking the ends of the integrated form of pHS87 were found near-identical to *attB* and *attP*, consistent with the integration scenario. The abundance of *attB*, *attP* and *attR* was estimated by quantitative PCR (S3 Fig) Results showed that the *tRNA*^*Thr*^ site was ~98% occupied by the integrated form of pHS87b. And the abundance of the circular form of pHS87b was ~2-fold higher than that of the integrated form, suggesting pHS87b might be capable of self-replication, though the replication genes are currently unknown. In accord to the above result, the Southern blotting assays showed pHS87b-specific probe bind to the plasmid and chromosome (S4 Fig).

Besides, a genomic island syntenic to the GI form of pHS87b was found at the same locus of the chromosome of *P*. *aeruginosa* NCGM 1900 (GenBank: AP014622) (Fig 2E). In addition, a chromosomal region syntenic to the GI form of pHS87b was also found in *P*. *aeruginosa* NCGM 1984 (GenBank: AP014646) (Fig 2E). Interestingly, this relevant region was 36-kb downstream of the *tRNA*^*Thr*^ gene but delimited by the same *tRNA*^*Thr*^-associated direct repeats. Phage-related genes lie between the *tRNA*^*Thr*^ gene and pHS87b syntenic region, possibly composing another prophage-type GI, suggesting a second insertion at the *tRNA*^*Thr*^ locus occurred and the pHS87b-syntenic region does have association with the *tRNA*^*Thr*^ locus. This evidence further supports the conclusion that pHS87b is *tRNA*^*Thr*^ site-specific integrative. tRNA genes have been shown to be hot spots for GI integration. Few cases of plasmid integration have been reported in *P*. *aeruginosa*. Two related plasmids pKLK106 and pKLC102 are known to coexist as a free plasmid and a genomic island in *P*. *aeruginosa* [4]. pKLC102 can be incorporated into the *tRNA*^*Lys*^ gene under the control of a phage-related XerC-like integrase via targeting the cognate attachment site, showing a similar manner to pHS87b. The integration feature of pHS87b also exhibits a potential to be used in genetic manipulation of *P*. *aeruginosa*.

### General features of pHS87a

The native plasmid pHS87a contains a backbone region and a variable region. Notably, within the variable region lies an *aacA4* gene that may confer resistance to aminoglycoside antibiotics.

Plasmid pHS87a bears a putative replicon nearly identical to that of the cryptic *Thiobacillus intermedius* plasmid pTiK12 (GenBank: L36865). The pHS87a plasmid possesses a *repA* gene and an AT-rich region located upstream that potentially can form a stem-loop structure similar to that proposed for pTiK1, but lacks the downstream region containing a series of tandem direct repeats presented in pTiK12. In pTiK12, *repA* and the upstream region are thought to be involved in replication, while the tandem repeats might have a regulation role in plasmid incompatibility [29]. Blastn search revealed that the replicon also is present in *Xanthomonas axonopodis* plasmids, including pXAC33 (GenBank: AE008924) and pXAC64 (GenBank: AE008925) [30]. In addition, the putative RepA protein of pHS87a shows significant similarity (60% – 70%) to the RepA of well-described pPT23A-family plasmids found in *Pseudomonas syringae* [31]. pPT23A-family plasmids encode diverse functions such as virulence and fitness. Therefore, those plasmids may be evolutionarily related (S5 Fig). pHS87a carries *parA* and *parC* that might be responsible for plasmid stability. Notably, pHS87a encodes a putative *relBE* toxin-antitoxin (TA) system. Plasmid-borne TA systems has been proved to be capable to maintain plasmid stability via post-segregational killing mechanism [32]. pHS87a also carries other genes likely involved in DNA processing, such as *orf19* and *orf20* coding for putative resolvase domain-containing protein and adenine-specific DNA methyltransferase, respectively. No conjugative modules were detected, indicating that pHS87a may not be a self-transmissible plasmid.

Plasmid pHS87a has a variable region with mobile elements closely adjacent to each other (Fig 1). It has a Tn*402*-like class 1 integron, into which an *aacA4* gene cassette is inserted. The *aacA4* gene encodes aminoglycoside N(6')-acetyltransferase-IIb which has been known to confer resistance to gentamycin, tobramycin and netilmicin, but not to amikacin due to a single amino acid change [33]. The putative integron has the 5’-conserved segment (5’-CS) but lacks the 3’-CS. It resides in a Tn*402* transposon which is flanked by 5-bp direct repeats (5’-AAACC-3’). The transposon encodes putative transposition genes *tniABQC* and has 25-bp imperfect inverted terminal repeats (IRs, 5’-TGTCgTTTTCAGAAGACGgCTGCAC-3’/5’-GTGCAGtCGTCTTCTGAAAAtGACA-3’). Tn*402*-like class 1 integron has been one of the major vehicles for a large set of antibiotic-resistant genes [34]. Its frequent detection including here highlights its important role in resistance dissemination. In addition, pHS87a carries a transposon Tn*6049* and a truncated IS element ΔIS*1071*. Tn*6049* has 12-bp imperfect inverted terminal repeats (5’-TGTGCaTaAGCA-3’/5’-TGCTcAcGCACA-3’) and is flanked by 4-bp direct repeats (5’-GCAG-3’). Tn*6049* has been found highly abundant as a promiscuous transposable element in the genome of *Cupriavidus metallidurans* CH34 [35], a strain widely used to study heavy metal-related cellular processes. The ΔIS*1071* element has only one terminus matching the 110-bp IR_R_ of IS*1071* and a truncated transposase gene. Interestingly, a genomic island in *C*. *metallidurans* CH34, CMGI-3 also has multiple copies of IS*1071* [35], further suggesting pHS87a might have acquired genetic materials from sources related to *C*. *metallidurans*. However, the segment between Tn*6049* and ΔIS*1071* has no homology to *C*. *metallidurans* CH34. The variable region comprises nearly 57% of the whole plasmid in length, indicating pHS87a is a plastic plasmid and may have evolved with multiple origins.

Furthermore, we mapped the nucleotide sequence data from the NCBI and HMP to that of pHS87a (S6 Fig) to analyse the distribution of the modules of the plasmid. The backbone of pHS87a has limited coverage in the NCBI database and no coverage in the HMP resource, suggesting the backbone has been rarely detected and may not be related to human microbiome. By contrast, the ΔIS*1071*, Tn*6049* and Tn*402*-like class 1 integron possess different levels of richness in the NCBI database, indicating genetic exchanges has promoted the amplification of mobile genetic elements. However, the Tn*6049* was not found in the HMP resource. In addition, pHS87a only shares a *kfrA*-like gene and integron-related structures with some of the sequenced *P*. *aeruginosa* plasmids and no other similarities.

## Conclusions

We completely sequenced two novel plasmids found in *P*. *aeruginosa* HS87. pHS87b is a phage-related site-specific integrative plasmid, while pHS87a is a plastic antibiotic-resistant plasmid. The two native plasmids may promote the fitness and evolution of the strain.

## Supporting Information

S1 Fig Coverage of the pHS87b sequence by different resources. Coverage of a position means the times the nucleotide acid at this position was aligned. HMP, Human Microbiome Project; nr, NCBI non-redundant database; Pa plasmid, completely sequenced *P*. *aeruginosa* plasmids.
(TIF)

S2 Fig Digestion profile of the GI occupying the *tRNA*^*Thr*^ gene site is consistent with the predicted profile of the integrated form of pHS87b. The amplicon (*) was purified. Sequence analysis of the integrated form of pHS87b was based on the sequence of the pHS87b and sequenced junctions. The size number in the table is placed in accord with the maker. Grey numbers are the sizes of predicted fragments not shown in the gel which may be due to low DNA concentrations.
(TIF)

S3 Fig Abundance of the empty *tRNA*^*Thr*^ site and the circular and integrated forms of pHS87b. The *attB*, *attP* and *attR* indicates the empty integration site, circular and integrated forms of pHS87b, respectively. The *gyrB* gene is used as endogenous reference. The fold change was calculated by 2-ΔΔCT, as the amplification efficiencies of different primers were about 100% and approximately equal to each other (data not shown).
(TIF)

S4 Fig Southern blotting to verify that pHS87b integrates into chromosome. The *pchG* gene on chromosome and the integrase gene *orf2* on pHS87b were used as the probes. Lane M: 1kb plus DNA ladder. A: 1% agarose gel electrophoresis of *P*. *aeruginosa* HS87 genomic DNA. B: Sothern blotting with pHS87b-specific probe. C: Sothern blotting with chromosome-specific probe.
(TIF)

S5 Fig Phylogenetic tree of RepA proteins of pHS87a and pPT23A-family plasmids. Plasmid names, host, the accession numbers of RepA proteins are given. Plasmids of pPT23A family were from Zhao *et al* [31].
(TIF)

S6 Fig Coverage of the pHS87a sequence by different resources. Coverage of a position means the times the nucleotide acid at this position was aligned. HMP, Human Microbiome Project; nr, NCBI non-redundant database; Pa plasmid, completely sequenced *P*. *aeruginosa* plasmids.
(TIF)

S1 Table Annotation of plasmid pHS87a. (DOC)

S2 Table Annotation of plasmid pHS87b. (DOC)

S3 Table Primers used in this study. (DOC)

## References

[1] Frost LS, Leplae R, Summers AO, Toussaint A. Mobile genetic elements: the agents of open source evolution. Nat Rev Microbiol. 2005;3(9):722–32. 16138100 10.1038/nrmicro1235

[2] Strateva T, Yordanov D. *Pseudomonas aeruginosa*—a phenomenon of bacterial resistance. J Med Microbiol. 2009;58(9):1133–48. 19528173 10.1099/jmm.0.009142-0

[3] Campbell AM. Chromosomal insertion sites for phages and plasmids. J Bacteriol. 1992;174(23):7495–9. 1447124 10.1128/jb.174.23.7495-7499.1992 PMC207458

[4] Klockgether J, Reva O, Larbig K, Tummler B. Sequence analysis of the mobile genome island pKLC102 of *Pseudomonas aeruginosa* C. J Bacteriol. 2004;186(2):518–34. 14702321 10.1128/JB.186.2.518-534.2004 PMC305764

[5] Korfhagen TR, Ferrel JA, Menefee CL, Loper JC. Resistance plasmids of *Pseudomonas aeruginosa*: change from conjugative to nonconjugative in a hospital population. Antimicrob Agents Chemother. 1976;9(5):810–6. 821390 10.1128/aac.9.5.810 PMC429626

[6] Pansegrau W, Lanka E, Barth PT, Figurski DH, Guiney DG, Haas D, et al Complete nucleotide sequence of Birmingham IncP alpha plasmids. Compilation and comparative analysis. J Mol Biol. 1994;239(5):623–63. 8014987 10.1006/jmbi.1994.1404

[7] Di Pilato V, Pollini S, Rossolini GM. Characterization of plasmid pAX22, encoding VIM-1 metallo-beta-lactamase, reveals a new putative mechanism of In70 integron mobilization. J Antimicrob Chemother. 2014;69(1):67–71. 10.1093/jac/dkt311 23928025

[8] Odumosu BT, Adeniyi BA, Chandra R. Analysis of integrons and associated gene cassettes in clinical isolates of multidrug resistant *Pseudomonas aeruginosa* from Southwest Nigeria. Ann Clin Microbiol Antimicrob. 2013;12:29 10.1186/1476-0711-12-29 24143920 PMC3842740

[9] Kobayashi K, Hayashi I, Kouda S, Kato F, Fujiwara T, Kayama S, et al Identification and characterization of a novel *aac(6')*-*Iag* associated with the *bla*_IMP-1_-integron in a multidrug-resistant *Pseudomonas aeruginosa*. PLoS One. 2013;8(8):e70557 10.1371/journal.pone.0070557 23950962 PMC3741272

[10] Xiong J, Alexander DC, Ma JH, Deraspe M, Low DE, Jamieson FB, et al Complete sequence of pOZ176, a 500-kilobase IncP-2 plasmid encoding IMP-9-mediated carbapenem resistance, from outbreak isolate *Pseudomonas aeruginosa* 96. Antimicrob Agents Chemother. 2013;57(8):3775–82. 10.1128/AAC.00423-13 23716048 PMC3719692

[11] Naas T, Bonnin RA, Cuzon G, Villegas MV, Nordmann P. Complete sequence of two KPC-harbouring plasmids from *Pseudomonas aeruginosa*. J Antimicrob Chemother. 2013;68(8):1757–62. 10.1093/jac/dkt094 23569197

[12] Jovcic B, Lepsanovic Z, Begovic J, Rakonjac B, Perovanovic J, Topisirovic L, et al The clinical isolate *Pseudomonas aeruginosa* MMA83 carries two copies of the *bla*_NDM-1_ gene in a novel genetic context. Antimicrob Agents Chemother. 2013;57(7):3405–7. 10.1128/AAC.02312-12 23612199 PMC3697382

[13] Haines AS, Jones K, Batt SM, Kosheleva IA, Thomas CM. Sequence of plasmid pBS228 and reconstruction of the IncP-1alpha phylogeny. Plasmid. 2007;58(1):76–83. 17320955 10.1016/j.plasmid.2007.01.001

[14] Haines AS, Jones K, Cheung M, Thomas CM. The IncP-6 plasmid Rms149 consists of a small mobilizable backbone with multiple large insertions. J Bacteriol. 2005;187(14):4728–38. 15995187 10.1128/JB.187.14.4728-4738.2005 PMC1169491

[15] Li H, Toleman MA, Bennett PM, Jones RN, Walsh TR. Complete Sequence of p07-406, a 24,179-base-pair plasmid harboring the *bla*_VIM-7_ metallo-beta-lactamase gene in a *Pseudomonas aeruginosa* isolate from the United States. Antimicrob Agents Chemother. 2008;52(9):3099–105. 10.1128/AAC.01093-07 18591274 PMC2533458

[16] Harrison EM, Carter ME, Luck S, Ou HY, He X, Deng Z, et al Pathogenicity islands PAPI-1 and PAPI-2 contribute individually and synergistically to the virulence of *Pseudomonas aeruginosa* strain PA14. Infect Immun. 2010;78(4):1437–46. 10.1128/IAI.00621-09 20123716 PMC2849418

[17] Ramirez-Diaz MI, Diaz-Magana A, Meza-Carmen V, Johnstone L, Cervantes C, Rensing C. Nucleotide sequence of *Pseudomonas aeruginosa* conjugative plasmid pUM505 containing virulence and heavy-metal resistance genes. Plasmid. 2011;66(1):7–18. 10.1016/j.plasmid.2011.03.002 21421005

[18] Yeldho D, Rebello S, Jisha MS. Plasmid-mediated biodegradation of the anionic surfactant sodium dodecyl sulphate, by *Pseudomonas aeruginosa* S7. Bull Environ Contam Toxicol. 2011;86(1):110–3. 10.1007/s00128-010-0162-2 21152890

[19] Delcher AL, Harmon D, Kasif S, White O, Salzberg SL. Improved microbial gene identification with GLIMMER. Nucleic Acids Res. 1999;27(23):4636–41. 10556321 10.1093/nar/27.23.4636 PMC148753

[20] Borodovsky M, Mills R, Besemer J, Lomsadze A. Prokaryotic gene prediction using GeneMark and GeneMark.hmm. Curr Protoc Bioinformatics. 2003;Chapter 4:Unit4 5. 10.1002/0471250953.bi0405s01 18428700

[21] Hyatt D, Chen GL, Locascio PF, Land ML, Larimer FW, Hauser LJ. Prodigal: prokaryotic gene recognition and translation initiation site identification. BMC Bioinformatics. 2010;11:119 10.1186/1471-2105-11-119 20211023 PMC2848648

[22] Tamura K, Peterson D, Peterson N, Stecher G, Nei M, Kumar S. MEGA5: molecular evolutionary genetics analysis using maximum likelihood, evolutionary distance, and maximum parsimony methods. Mol Biol Evol. 2011;28(10):2731–9. 10.1093/molbev/msr121 21546353 PMC3203626

[23] Edgar RC. MUSCLE: multiple sequence alignment with high accuracy and high throughput. Nucleic Acids Res. 2004;32(5):1792–7. 15034147 10.1093/nar/gkh340 PMC390337

[24] Ou HY, Chen LL, Lonnen J, Chaudhuri RR, Thani AB, Smith R, et al A novel strategy for the identification of genomic islands by comparative analysis of the contents and contexts of tRNA sites in closely related bacteria. Nucleic Acids Res. 2006;34(1):e3 16414954 10.1093/nar/gnj005 PMC1326021

[25] Roy PH, Tetu SG, Larouche A, Elbourne L, Tremblay S, Ren Q, et al Complete genome sequence of the multiresistant taxonomic outlier *Pseudomonas aeruginosa* PA7. PLoS One. 2010;5(1):e8842 10.1371/journal.pone.0008842 20107499 PMC2809737

[26] Fouts DE. *Phage_Finder*: automated identification and classification of prophage regions in complete bacterial genome sequences. Nucleic Acids Res. 2006;34(20):5839–51. 17062630 10.1093/nar/gkl732 PMC1635311

[27] Boyd EF, Almagro-Moreno S, Parent MA. Genomic islands are dynamic, ancient integrative elements in bacterial evolution. Trends Microbiol. 2009;17(2):47–53. 10.1016/j.tim.2008.11.003 19162481

[28] Stover CK, Pham XQ, Erwin AL, Mizoguchi SD, Warrener P, Hickey MJ, et al Complete genome sequence of *Pseudomonas aeruginosa* PAO1, an opportunistic pathogen. Nature. 2000;406(6799):959–64. 10984043 10.1038/35023079

[29] English RS, Lorbach SC, Huffman KM, Shively JM. Isolation and characterization of the replicon of a *Thiobacillus intermedius* plasmid. Plasmid. 1995;33(1):1–6. 7753904 10.1006/plas.1995.1001

[30] da Silva AC, Ferro JA, Reinach FC, Farah CS, Furlan LR, Quaggio RB, et al Comparison of the genomes of two *Xanthomonas* pathogens with differing host specificities. Nature. 2002;417(6887):459–63. 12024217 10.1038/417459a

[31] Zhao Y, Ma Z, Sundin GW. Comparative genomic analysis of the pPT23A plasmid family of *Pseudomonas syringae*. J Bacteriol. 2005;187(6):2113–26. 15743960 10.1128/JB.187.6.2113-2126.2005 PMC1064049

[32] Shao Y, Harrison EM, Bi D, Tai C, He X, Ou HY, et al TADB: a web-based resource for Type 2 toxin-antitoxin loci in bacteria and archaea. Nucleic Acids Res. 2011;39(suppl 1): D606–11. 20929871 10.1093/nar/gkq908 PMC3013778

[33] Rather PN, Munayyer H, Mann PA, Hare RS, Miller GH, Shaw KJ. Genetic analysis of bacterial acetyltransferases: identification of amino acids determining the specificities of the aminoglycoside 6'-N-acetyltransferase Ib and IIa proteins. J Bacteriol. 1992;174(10):3196–203. 1577689 10.1128/jb.174.10.3196-3203.1992 PMC205986

[34] Gillings MR. Integrons: past, present, and future. Microbiol Mol Biol Rev. 2014;78(2):257–77. 10.1128/MMBR.00056-13 24847022 PMC4054258

[35] Ricker N, Qian H, Fulthorpe RR. The limitations of draft assemblies for understanding prokaryotic adaptation and evolution. Genomics. 2012;100(3):167–75. 10.1016/j.ygeno.2012.06.009 22750556

