# Supplementary figures and images for: A Site-Specific Integrative Plasmid Found in Pseudomonas aeruginosa Clinical Isolate HS87 along with A Plasmid Carrying an Aminoglycoside-Resistant Gene
Source: PLoS One. 2016 Feb 3;11(2):e0148367. doi: 10.1371/journal.pone.0148367 (PMC4739549; doi:10.1371/journal.pone.0148367)

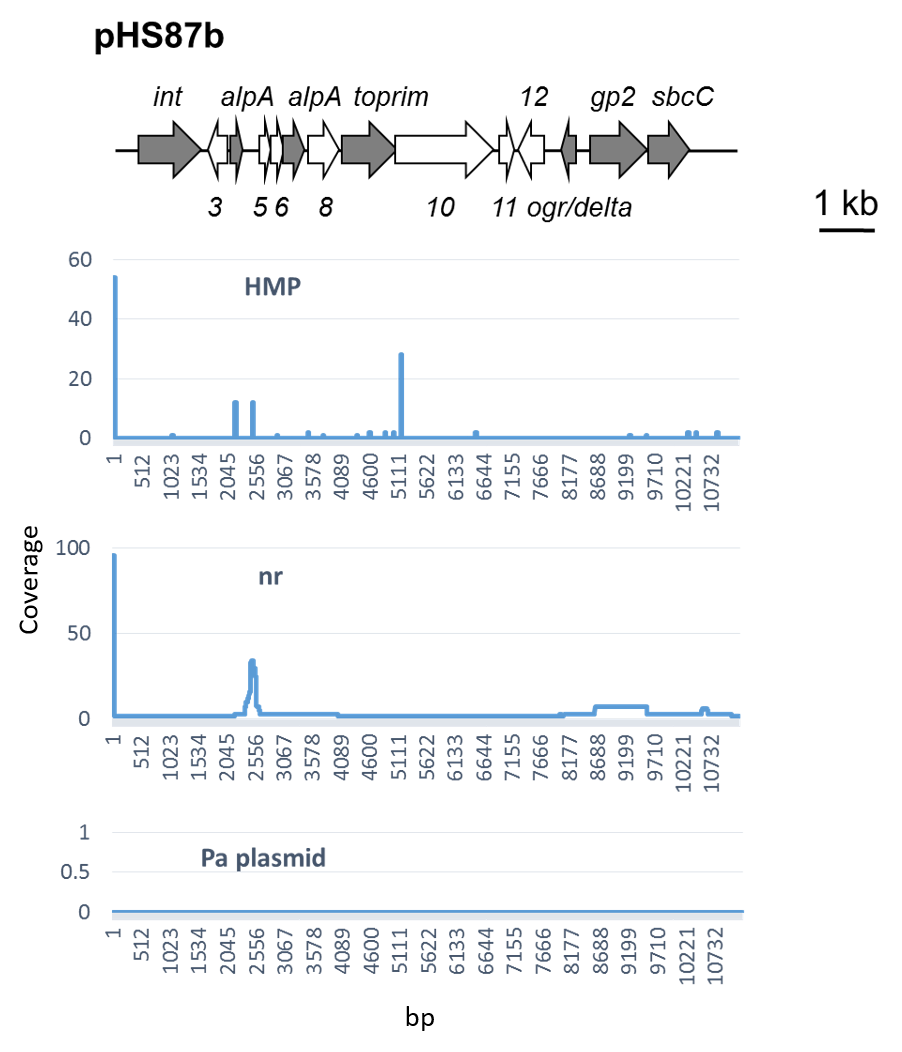

Supplement: S1 Fig — Coverage of a position means the times the nucleotide acid at this position was aligned. HMP, Human Microbiome Project; nr, NCBI non-redundant database; Pa plasmid, completely sequenced P. aeruginosa plasmids. (TIF) [file pone.0148367.s001.tif]

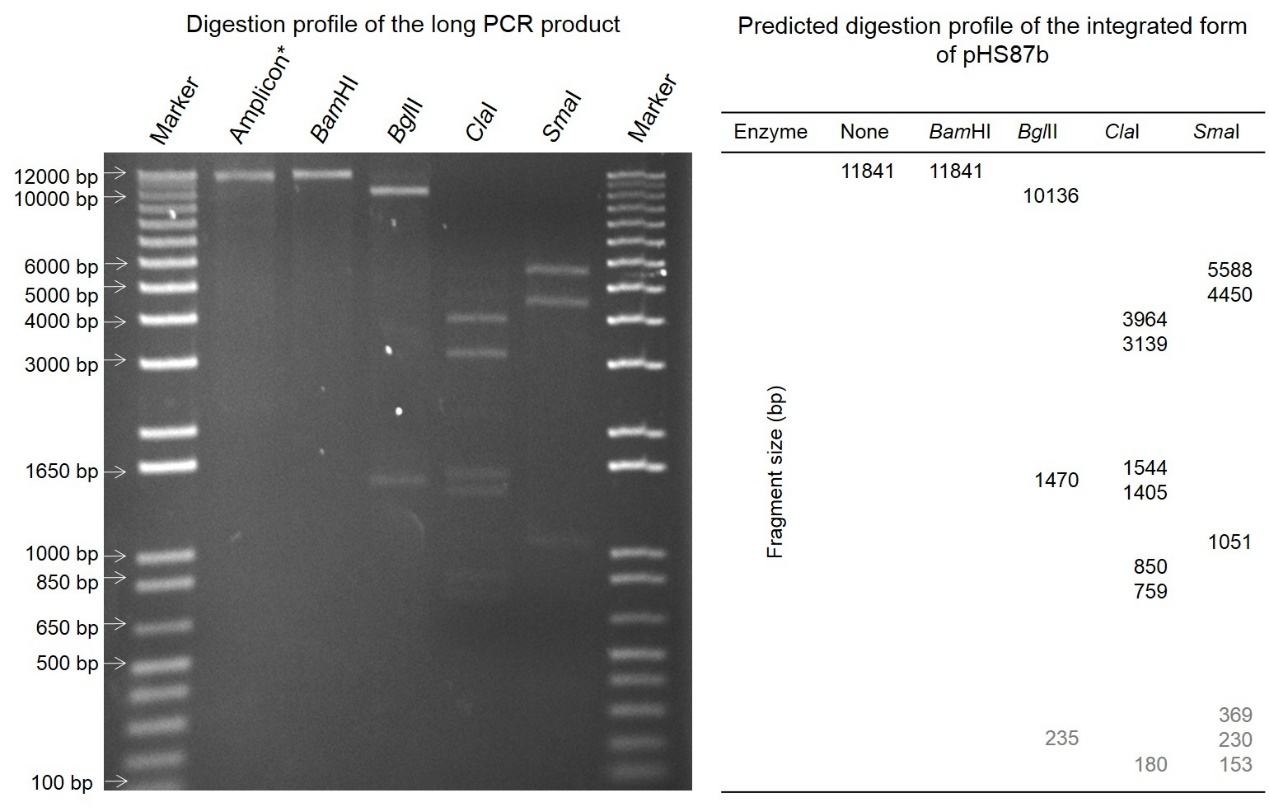

Supplement: S2 Fig — The amplicon (*) was purified. Sequence analysis of the integrated form of pHS87b was based on the sequence of the pHS87b and sequenced junctions. The size number in the table is placed in accord with the maker. Grey numbers are the sizes of predicted fragments not shown in the gel which may be due to low DNA concentrations. (TIF) [file pone.0148367.s002.tif]

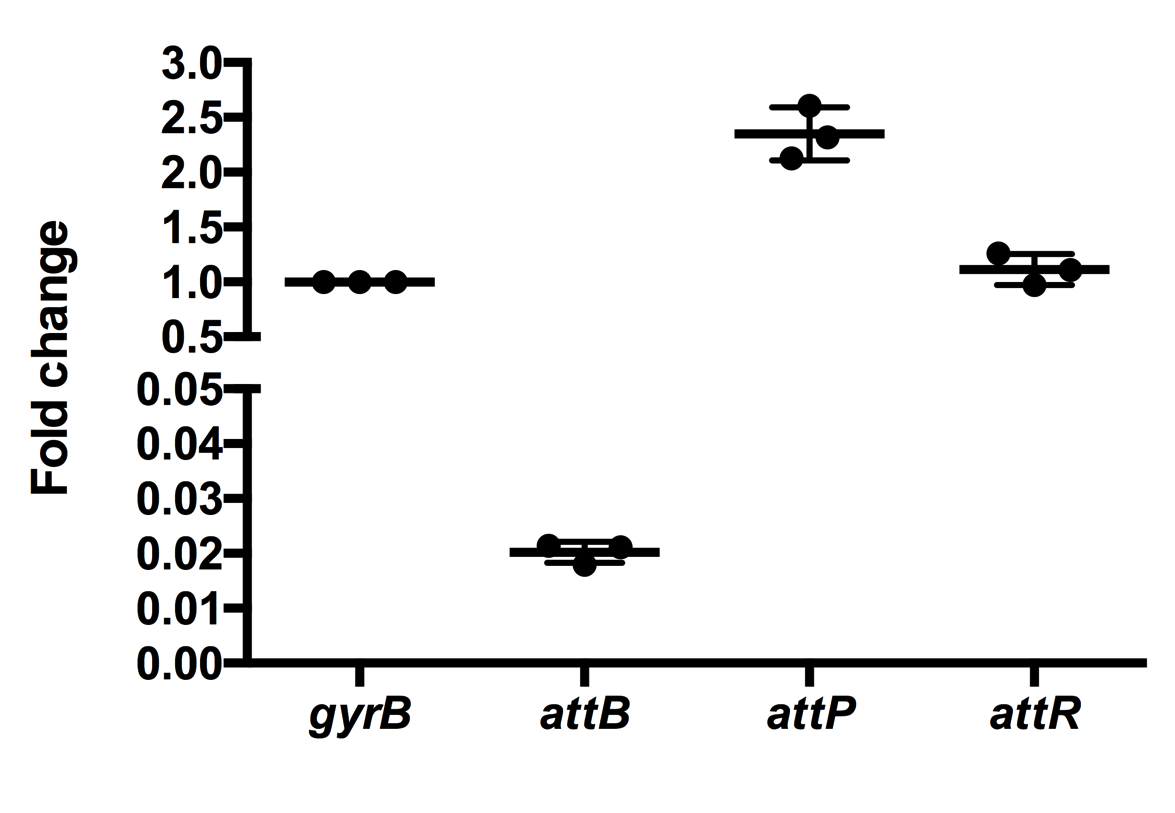

Supplement: S3 Fig — The attB, attP and attR indicates the empty integration site, circular and integrated forms of pHS87b, respectively. The gyrB gene is used as endogenous reference. The fold change was calculated by 2-ΔΔCT, as the amplification efficiencies of different primers were about 100% and approximately equal to each other (data not shown). (TIF) [file pone.0148367.s003.tif]

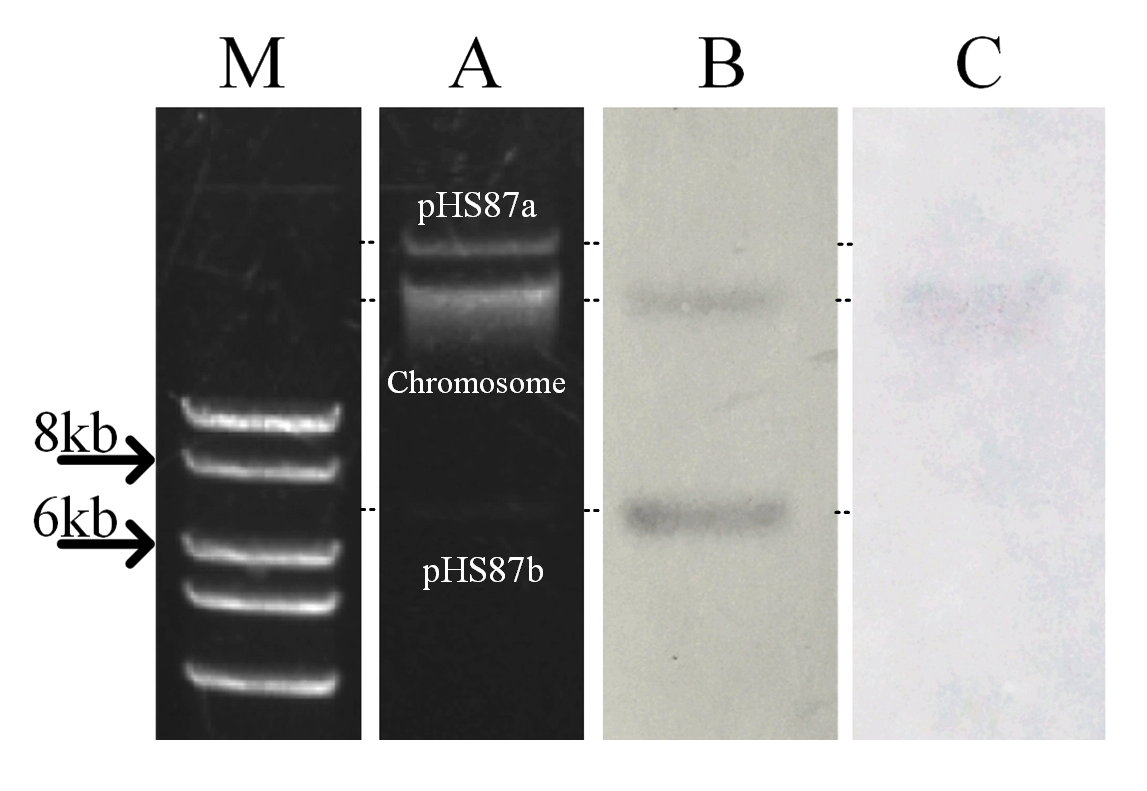

Supplement: S4 Fig — The pchG gene on chromosome and the integrase gene orf2 on pHS87b were used as the probes. Lane M: 1kb plus DNA ladder. A: 1% agarose gel electrophoresis of P. aeruginosa HS87 genomic DNA. B: Sothern blotting with pHS87b-specific probe. C: Sothern blotting with chromosome-specific probe. (TIF) [file pone.0148367.s004.tif]

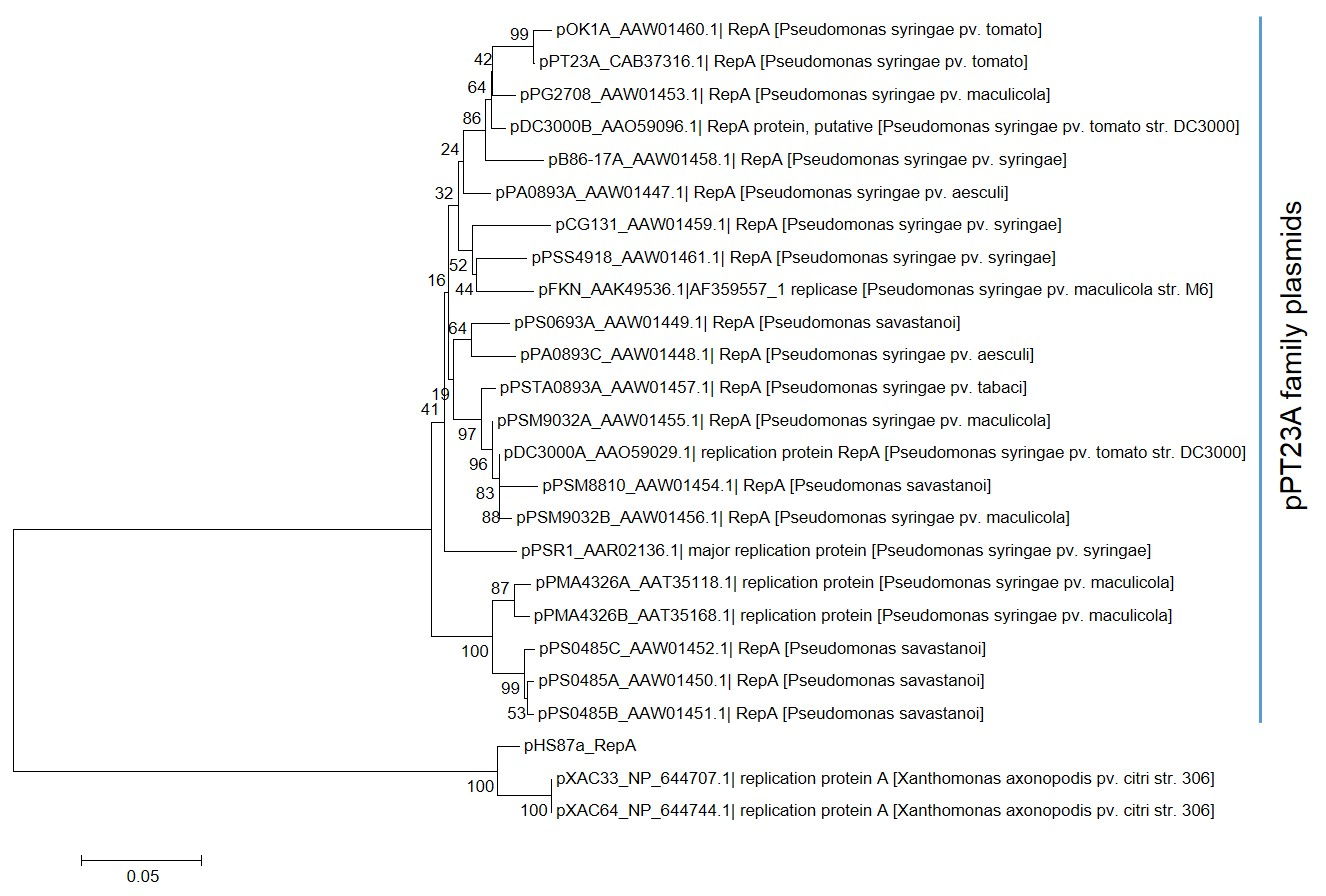

Supplement: S5 Fig — Plasmid names, host, the accession numbers of RepA proteins are given. Plasmids of pPT23A family were from Zhao et al [31]. (TIF) [file pone.0148367.s005.tif]

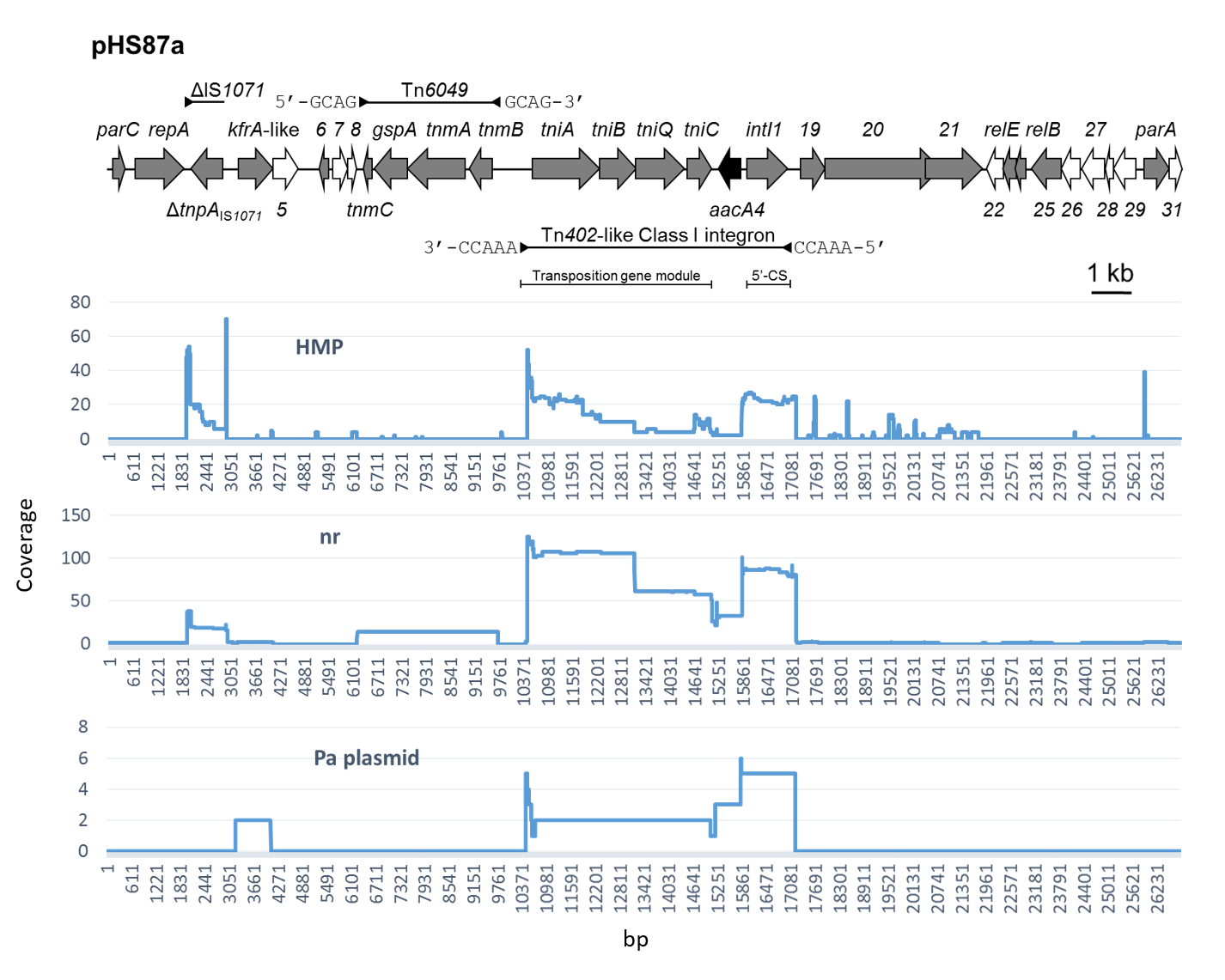

Supplement: S6 Fig — Coverage of a position means the times the nucleotide acid at this position was aligned. HMP, Human Microbiome Project; nr, NCBI non-redundant database; Pa plasmid, completely sequenced P. aeruginosa plasmids. (TIF) [file pone.0148367.s006.tif]
