# Supplementary material for: A Site-Specific Integrative Plasmid Found in Pseudomonas aeruginosa Clinical Isolate HS87 along with A Plasmid Carrying an Aminoglycoside-Resistant Gene
Source: PLoS One. 2016 Feb 3;11(2):e0148367. doi: 10.1371/journal.pone.0148367 (PMC4739549; doi:10.1371/journal.pone.0148367)
Supplement: S1 Table — (DOC) [file pone.0148367.s007.doc]

**S1 Table. Annotation of plasmid pHS87a.**

| **ORF** | **Gene** | **Left end** | **Right end** | **Strand** | **Length** | **No. of aa** | **Product** |
| --- | --- | --- | --- | --- | --- | --- | --- |
| *orf1* | *parC* | 148 | 459 | + | 312 | 103 | Partition protein C |
| *orf2* | *repA* | 706 | 1926 | + | 1221 | 406 | Plasmid replicase RepA |
| *orf3* | *tnpA* | 2096 | 2896 | - | 801 | 266 | Truncated IS*1071* transposase |
| *orf4* | *kfrA* | 3288 | 4160 | + | 873 | 290 | KfrA-like protein |
| *orf5* | - | 4147 | 4776 | + | 630 | 209 | Hypothetical protein |
| *orf6* | - | 5303 | 5554 | - | 252 | 83 | Helix-turn-helix domain protein |
| *orf7* | - | 5641 | 6018 | + | 378 | 125 | Hypothetical protein |
| *orf8* | - | 6015 | 6221 | + | 207 | 68 | Hypothetical protein |
| *orf9* | *tnmC* | 6400 | 6615 | - | 216 | 71 | Hypothetical protein |
| *orf10* | *gspA* | 6669 | 7502 | - | 834 | 277 | General secretion pathway ATPase |
| *orf11* | *tnmA* | 7499 | 8938 | - | 1440 | 479 | Tn*6049* transposase |
| *orf12* | *tnmB* | 9050 | 9619 | - | 570 | 189 | Tn*6049* mobility protein |
| *orf13* | *tniA* | 10617 | 12296 | + | 1680 | 559 | TniA |
| *orf14* | *tniB* | 12299 | 13207 | + | 909 | 302 | TniB |
| *orf15* | *tniQ* | 13204 | 14421 | + | 1218 | 405 | TniQ |
| *orf16* | *tniC* | 14483 | 15106 | + | 624 | 207 | TniC |
| *orf17* | *aacA4* | 15269 | 15823 | - | 555 | 184 | Aminoglycoside 6’-N acetyltransferase AAC(6’)-IId |
| *orf18* | *intI1* | 15984 | 16997 | + | 1014 | 337 | IntI1 integrase |
| *orf19* | - | 17314 | 17919 | + | 606 | 201 | Resolvase domain-containing protein |
| *orf20* | - | 17916 | 20798 | + | 2883 | 960 | Adenine specific DNA methyltransferase |
| *orf21* | - | 20420 | 21853 | + | 1434 | 477 | Major facilitator transporter |
| *orf22* | - | 21945 | 22364 | - | 420 | 139 | Hypothetical protein |
| *orf23* | *relE* | 22361 | 22690 | - | 330 | 109 | Addiction module toxin, RelE/StbE family |
| *orf24* | *relB* | 22662 | 22943 | - | 282 | 93 | Addiction module antitoxin, RelB/DinJ family |
| *orf25* | - | 23072 | 23824 | - | 753 | 250 | Exonuclease RNase T and DNA polymerase III |
| *orf26* | - | 23821 | 24291 | - | 471 | 156 | Hypothetical protein |
| *orf27* | - | 24315 | 24884 | - | 570 | 189 | HAD-superfamily hydrolase, subfamily IA, variant 3 |
| *orf28* | - | 24917 | 25120 | - | 204 | 67 | Hypothetical protein |
| *orf29* | - | 25122 | 25685 | - | 564 | 187 | Hypothetical protein |
| *orf30* | *parA* | 25879 | 26505 | + | 627 | 208 | Putative partition protein |
| *orf31* | - | 26502 | 26825 | + | 324 | 107 | Hypothetical protein |
