# Supplementary material for: A Site-Specific Integrative Plasmid Found in Pseudomonas aeruginosa Clinical Isolate HS87 along with A Plasmid Carrying an Aminoglycoside-Resistant Gene
Source: PLoS One. 2016 Feb 3;11(2):e0148367. doi: 10.1371/journal.pone.0148367 (PMC4739549; doi:10.1371/journal.pone.0148367)
Supplement: S2 Table — (DOC) [file pone.0148367.s008.doc]

**S2 Table. Annotation of plasmid pHS87b.**

| **ORF** | **Gene** | **Left end** | **Right end** | **Strand** | **Length** | **No. of aa** | **Product** |
| --- | --- | --- | --- | --- | --- | --- | --- |
| *attP* |  | 1 | 45 |  |  |  |  |
| *orf2* | *int* | 309 | 1565 | + | 1257 | 418 | Integrase family protein |
| *orf3* | - | 1681 | 2034 | - | 455 | 147 | Hypothetical protein |
| *orf4* | *alpA* | 2097 | 2312 | + | 216 | 71 | AlpA family transcriptional regulator |
| *orf5* | - | 2624 | 2824 | + | 201 | 66 | Hypothetical protein |
| *orf6* | - | 2821 | 3048 | + | 228 | 75 | Hypothetical protein |
| *orf7* | *alpA* | 3045 | 3431 | + | 387 | 128 | AlpA family transcriptional regulator |
| *orf8* | - | 3496 | 4062 | + | 567 | 188 | Hypothetical protein |
| *orf9* | *toprim* | 4059 | 5069 | + | 1011 | 336 | TOPRIM domain-containing protein |
| *orf10* | - | 5066 | 6853 | + | 1788 | 595 | Hypothetical protein |
| *orf11* | - | 6945 | 7238 | + | 294 | 97 | Hypothetical protein |
| *orf12* | - | 7279 | 7758 | - | 480 | 159 | Hypothetical protein |
| *orf13* | *ogr* | 8066 | 8338 | - | 273 | 90 | Phage transcriptional activator Ogr/delta |
| *orf14* | *gp2* | 8593 | 9618 | + | 1026 | 341 | P2 family phage major capsid protein |
| *orf15* | *sbcC* | 9641 | 10393 | + | 753 | 250 | Exonuclease SbcC |

*attP* is not an open reading frame.
