# Supplementary material for: A Site-Specific Integrative Plasmid Found in Pseudomonas aeruginosa Clinical Isolate HS87 along with A Plasmid Carrying an Aminoglycoside-Resistant Gene
Source: PLoS One. 2016 Feb 3;11(2):e0148367. doi: 10.1371/journal.pone.0148367 (PMC4739549; doi:10.1371/journal.pone.0148367)
Supplement: S3 Table — (DOC) [file pone.0148367.s009.doc]

**S3 Table. Primers used in this study.**

| **Name** | **Target** | **Sequence (5’ to 3’)** |
| --- | --- | --- |
| thr_U | *tRNA^Thr^* gene | ACTACTTCACCATGCTCGGC |
| thr_D |  | GTAGGTGAGCTTGTCGAGGC |
| attP_U | *attP* of pHS87b | GCACCGCACTACGCTGA |
| attP_D |  | CACGAATCACGCCAACATAC |
| int-SBF | integrase gene *orf2* on pHS87b | CCGCCTCTGTCATTTCGATG |
| int-SBR |  | GTGCGCAGATATTAGCGAGG |
| pchG-SBF | *pchG* gene on chromosome | ATGAGCGACGTCCGTTCCGTG |
| pchG-SBR |  | GGTCACGAGGCTTGCTCCAGC |
| attB-qF | *attB* site on chromosome | TTCGGGCAGGAAAATCAGTG |
| attB-qR |  | GCATCTGACCCGGCATTCTA |
| attP-qF | *attP* of pHS87b | CGTTGACCTGGACATTGACC |
| attP-qR |  | CGCTGAAACACCAAGGAACA |
| gyrB-qF | *gyrB* gene on chromosome | GGCGTGGGTGTGGAAGTC |
| gyrB-qR |  | TGGTGGCGATCTTGAACTTCTT |
